# Patient Blood Management (PBM) as a Pillar in Surgical Safety and Care

**DOI:** 10.1590/0100-6991e-20250042EDIT01-en

**Published:** 2026-02-15

**Authors:** ISABEL CRISTINA CÉSPEDES, RAMIRO COLLEONI, NELSON AMERICO HOSSNE, MANOEL ANTONIO DE PAIVA, FRANZ ROBERT APODACA TORREZ, BARBARA BURZA BENIN, PIERRE FRANCOIS GEORGES SCHIPPERS, SHERRI OZAWA

**Affiliations:** 1 - Universidade Federal de São Paulo, Escola Paulista de Medicina - Disciplina de Genética, Departamento de Morfologia e Genética - São Paulo - SP - Brasil; 2 - Universidade Federal de São Paulo, Escola Paulista de Medicina - Disciplina de Gastroenterologia Cirúrgica, Departamento de Cirurgia- São Paulo - SP - Brasil; 3 - Universidade Federal de São Paulo, Escola Paulista de Medicina - Disciplina de Cirurgia Cardiovascular, Departamento de Cirurgia - São Paulo - SP - Brasil; 4 - Universidade Federal de São Paulo, Escola Paulista de Medicina - Disciplina de Neurocirurgia, Departamento de Neurologia e Neurocirurgia - São Paulo - SP - Brasil; 5 - Universidade Federal de São Paulo, Escola Paulista de Medicina - Departamento de Oncologia Clínica e Experimental - São Paulo - SP - Brasil; 6 - Society for the Advancement of Patient Blood Management (SABM) - Mount Royal - New Jersey - USA; 7 - Institute for Patient Blood Management and Bloodless Medicine and Surgery, Englewood Health - Englewood - New Jersey - USA

## INTRODUCTION

The Joint Commission International hospital accreditor, after evaluating medical records based on the potential risks and benefits of blood transfusions, published in 2023 that only 14% of blood component transfusions in the American hospitals studied were appropriate or adequately prescribed (Jadwin et al., 2023). This is because its effects are associated with a significant increase in mortality, infection, length of hospital stay, atrial fibrillation, kidney disorders (Blumberg et al., 2024; Cata et al., 2025), venous thromboembolism (Lin et al., 2020), and increased recurrence in cancer patients (Blumberg et al., 2024; Cata et al., 2025), even after excluding confounding factors such as comorbidities, disease severity, gender, age, and surgical team. This characterizes the transfusion of blood components as an independent risk factor for the aforementioned effects. 

Direct reactions to blood component transfusions, which range from fever and pruritus to severe situations such as dyspnea and anaphylactic shock, have a low prevalence, generally between 0.2% and 0.7% (SHOT, 2015; FDA, 2021; ANVISA, 2022; Davies et al., 2023; Public Health Agency of Canada, 2023; Samukange et al., 2023; Berg et al., 2024; Chen et al., 2025). 

However, blood transfusions are indirectly associated with the clinical repercussions described above through their inflammatory and immunosuppressive potential (Vargas et al., 2014). Recent studies have shown that blood bags contain bioactive molecules, such as microRNAs (miRNAs), mitochondrial DNA, cytokines, chemokines, procoagulant factors, and factors that regulate vascular dynamics, among others, with significant inflammatory and immunosuppressive effects (Almizraq et al., 2016; Said et al., 2018; Bagheri et al., 2024; Zhang et al., 2024). These bioactive molecules are present in blood components such as red blood cell concentrates, platelet concentrates, and fresh frozen plasma. 

In the context of inflammation, mitochondrial DNA released as cells break down in blood bags, plays a significant role. These molecules activate mechanisms that link mitochondrial stress to systemic inflammation, resulting in the production of pro-inflammatory cytokines and interferons (Bagheri et al., 2024). In addition, a recent study has shown that stored blood bags have twice as many inflammatory factors as fresh blood, due to the decomposition of its cellular components. These factors include IL-1β, IL-6, IL-12, and TNF-α, as well as miRNAs such as miR-33a-5p, which activates M1-type macrophages and increases the release of inflammatory factors (Zhang et al., 2024). In the immunological context, the effects include lymphopenia, modification of helper T cells, and activation of other immune cells (Vamvakas and Blajchman, 2007). In fact, blood transfusions represent a tissue transplant, with their repercussions (Nilsson et al., 2020). 

Therefore, scientific evidence has been increasingly strengthened in favor of the implementation of Patient Blood Management (PBM) programs. This program aims to establish a new line of care for clinical or surgical patients. In general, this program has three pillars. The 1st Pillar is due preparation of patients for events such as surgical procedures. This includes treating anemia and coagulation dysfunctions, as well as increasing patients’ blood cell reserves. The 2nd Pillar aims to avoid patients’ blood loss with more conservative surgical practices, effective use of hemostatics, optimization of blood collections for diagnostic testing, and recovery of the patient’s blood at the surgical site through intraoperative blood recovery equipment or even acute normovolemic hemodilution. In the 3rd. Pillar the aim is to make good use of the patients’ physiological reserves in tolerance to anemia, with a concept change for decision-making in the prescription of blood transfusions, from the use of only the hemoglobin index for a global clinical evaluation (Céspedes et al., 2024). There are at least 38 strategies for new and better care (Farmer et al., 2013). This new paradigm in transfusion practice is not fully integrated into medical school curricula (Al-Riyami et al., 2020), which makes its implementation a challenge due to the lack of knowledge and training, in addition to the necessary change in mentality.

PBM is a health policy urgently recommended by the World Health Organization - WHO (WHO, 2021; WHO, 2024). In Australia, its adoption resulted in a reduction in the use of packed red blood cells (41%), plasma (47%), and platelets (27%), with a 28% drop in mortality, 31% in the rate of infarction/stroke, 15% in length of hospital stay and 21% in infections, in addition to savings of US$ 100 million in six years in direct and indirect costs (Leahy et al., 2017). In Canada, the implementation of PBM, with recent publication of data from 20 years of success, has shown a significant reduction in infection rates and length of stay to less than half, saving about 50 million dollars per year (Pavenski et al., 2022). Other countries also report success stories, as in the case of Brazil, where the Paulista School of Medicine of the Federal University of São Paulo (UNIFESP) adopted extensive education programs for undergraduate and medical residency students on PBM, and established a program for the implementation of PBM in the line of patient care at Hospital São Paulo (HU/UNIFESP), as highlighted by WHO in the Guidance on Implementing Patient Blood Management (WHO, 2024). Researchers from the Paulista School of Medicine recently obtained an international award for a study of the epigenetic effects of blood transfusions, still unpublished in the scientific literature, which highlights the strength of molecular analyses in the field of blood transfusions (https://www.youtube.com/watch?v=UYB_hD2tLf4).

In a surgical patient, any additional agent that exacerbates the inflammatory response has short-, medium-, and long-term negative repercussions. In the short term, there is a predisposition to infectious complications due to higher levels of interleukin 6 (IL-6) or C-reactive protein (CRP) in the immediate postoperative period, which increases the risk of infection; in major surgeries, it can exacerbate the risks of cardiovascular, pulmonary, hepatic, and renal dysfunctions, including complications such as respiratory distress and renal failure; it can increase edema and pain with greater discomfort and the need for analgesia; there is a delay in healing through the production of free radicals and proteolytic mediators that interfere with the tissue matrix, including suture dehiscence (Watt et al., 2015). In the medium and long term, inflammatory repercussions correlate with greater need for intensive care and longer hospital stays; postoperative recovery is slower, with longer functional limitation and greater fatigue; inflammation is also associated with increased mortality (Bain et al., 2023; Cata et al., 2017). What about the immunosuppressive repercussions of blood transfusions? The increased risk of surgical site infection, nosocomial, sepsis, and other opportunistic infections becomes a real problem, increases the chance of complications like wound dehiscence, fistulas, and even necrosis. In addition, the attenuated immune response can mask signs and symptoms, delaying appropriate treatment, leading to longer hospitalization time and higher mortality (Sawyer and Claridge, 2007; Uçkay et al., 2009; Fishman, 2011). Implementing PBM is not just a paradigm shift in the prescription of blood products, but a matter of safety and ethics.
